# Effects of 3-nitrooxypropanol on methane emission, growth and feed intake in growing calves from 5 months of age

**DOI:** 10.1093/jas/skaf379

**Published:** 2025-10-30

**Authors:** Eline E A Burgers, André Bannink, Nicola Walker, Reto Zihlmann, Sanne Van Gastelen

**Affiliations:** Wageningen Livestock Research, Wageningen University & Research, Wageningen, 6700 AH, the Netherlands; Wageningen Livestock Research, Wageningen University & Research, Wageningen, 6700 AH, the Netherlands; dsm-firmenich, Wurmisweg 576, Kaiseraugst, 4303, Switzerland; dsm-firmenich, Wurmisweg 576, Kaiseraugst, 4303, Switzerland; Wageningen Livestock Research, Wageningen University & Research, Wageningen, 6700 AH, the Netherlands

**Keywords:** enteric methane production, feed additive, growing calf, methanogen inhibitor

## Abstract

This study aimed to test the effect of 3-nitrooxypropanol (**3-NOP**) on methane (**CH_4_**) emission, feed intake, and body weight (**BW**) of calves from 5 mo of age. Seventy calves were enrolled in the acclimatization period (3 wk), after which 60 calves were selected based on their ability to use the automated feed bins and the GreenFeed system. Calves (*N* = 60), a mixture of males and females and either Holstein Friesian or Belgian Blue, were blocked in pairs by sex, breed, and BW, and within block randomly assigned to 1 of 2 dietary treatments: a partially mixed ration (**PMR**) supplemented with (1) a placebo concentrate (**CTL**), or with (2) a 3-NOP concentrate (on average 121 mg 3-NOP/kg dry matter of total ration). The acclimatization period was followed by a baseline measurement period (2 wk), and afterwards the treatment period (12 wk) started where the calves received 1 of the 2 dietary treatments. The calves also received up to 1,280 g/day bait in the GreenFeed system, which was used for emission measurements. For analyses, all data were averaged for the baseline measurement period and for different periods in the treatment wk: 1–3, 4–6, 7–9 and 10–12. Upon 3-NOP supplementation, CH_4_ production (g/d) was reduced by 31.3% in wk 1–3, by 27.1% in wk 4–6, by 30.0% in wk 7–9, and by 30.7% in wk 10–12 compared to CTL (*P* < 0.01), and CH_4_ intensity (g/kg BW) was reduced by 30.0% in week 1–3, by 25.8% in wk 4–6, by 27.9% in wk 7–9, and by 28.6% in wk 10–12 compared to CTL (*P* < 0.01). Over the entire treatment period, 3-NOP calves had a reduction of 29.8% for CH_4_ production (*P* < 0.01), 19.4% for CH_4_ yield (g/kg dry matter intake; **DMI**) (*P* < 0.01), and 27.9% for CH_4_ intensity (g/kg BW) (*P* < 0.01). Calves receiving 3-NOP had a lower total DMI (6.1 kg/d) compared to CTL (6.9 kg/d) (*P* < 0.01), driven by a lower DMI of PMR. In every period, 3-NOP calves had a lower BW compared to CTL (over the entire treatment period: 251 vs. 258 kg, *P* < 0.01). The 3-NOP group and the CTL group did not differ in initial BW, but calves in the 3-NOP group had a lower final BW (290 kg) compared to CTL (301 kg) (*P* < 0.01). Despite the decreased DMI and growth performance, gain-to-feed ratio (kg BW gain/kg DMI) did not differ between the treatment groups. It can be concluded that 3-NOP persistently decreases CH_4_ emission in growing beef calves from 5 to 8 mo of age, but that it takes a longer period of time to grow the cattle to a similar body weight due to the decreased growth performance.

## Teaser

Supplementing 3-NOP to growing calves persistently reduced methane emission for at least 3 mo. Growing calves receiving 3-NOP in a starch-rich diet had a lower feed intake and body weight, although 3-NOP did not affect calves’ gain-to-feed ratio.

## Introduction

Cattle produce enteric methane (**CH_4_**) as a natural by-product of microbial fermentation in the rumen. This process allows them to effectively turn human inedible biomass, such as grass, into high quality protein in the form of milk and meat for human consumption (Gerber et al., 2015). Enteric CH_4_ emission contributes to greenhouse gas (**GHG**) emissions, and estimates suggest that the non-lactating and non-dairy cattle sector are the largest animal source of enteric CH_4_ emissions in Europe (FAOSTAT, 2019). Hence, enteric CH_4_ emission has become one of the main targets of GHG mitigation practices for the beef industry. Several dietary mitigation strategies exist to reduce enteric CH_4_ emission (Hristov et al., 2013). One of these strategies is a feed additive, 3-nitrooxypropanol (**3-NOP**; marketed as Bovaer, dsm-firmenich AG, Kaiseraugst, Switzerland). Arndt et al. (2022) reported that rumen manipulation by feeding 3-NOP was the most effective mitigation strategy to realize absolute reductions in CH_4_ emission. According to meta-analyses, 3-NOP effectively reduced enteric CH_4_ emissions in beef cattle by on average 22% to 35% (Dijkstra et al., 2018; Orzuna-Orzuna et al., 2024), which is a lower average reduction than reported for dairy cattle (39%; Dijkstra et al., 2018).

In dairy cows, efficacy of 3-NOP has been tested extensively, in many studies representing different stages of lactation (e.g. Melgar et al., 2021; Schilde et al., 2021; van Gastelen et al., 2024). Also, several studies investigated the efficacy of 3-NOP for beef cattle in feedlots (e.g. Kim et al., 2019; Alemu et al., 2021; Almeida et al., 2023, Orzuna-Orzuna et al., 2024). In the meta-analysis by Orzuna-Orzuna et al. (2024), the average body weight (**BW**) of the animals in the 15 included studies was 420 kg, which typically corresponds to approximately 12 mo of age in intensively reared beef cattle, though this may vary with nutrition and breed. It can be questioned whether younger calves respond differently upon 3-NOP supplementation. Calves are non-functional ruminants at birth and from 2 days of age, microorganisms start colonizing the rumen of the calves (Rey et al., 2014; Li et al., 2023). From weaning at 14 wk of age until 60 wk of age, enteric CH_4_ production increased progressively (Meale et al., 2021) as solid feed intake stimulates rumen development. Although calves at 5 mo already consume substantial amounts of solid feed and produce measurable CH_4_, their physiological response to 3-NOP cannot be assumed to be identical to that of older beef cattle as included in the meta-analysis by Orzuna-Orzuna et al. (2024), because differences in intake capacity, rumen development, and energy requirements may alter their sensitivity to 3-NOP. For young growing calves (i.e. <6 mo of age), studies of 3-NOP as a dietary additive to mitigate enteric CH_4_ emission are limited. When 3-NOP was supplied in early life, ie from birth until 3 wk post weaning, calves showed a persistent reduction in CH_4_ up to 12 mo of age (Meale et al., 2021). Only Kirwan et al. (2024) evaluated the effect of 3-NOP on enteric CH_4_ emission and performance in growing calves of less than 6 mo of age and concluded that CH_4_ emission was reduced by approximately 30%. Supplementation of 3-NOP did not affect dry matter intake (**DMI**), BW, or average daily gain (**ADG**). However, some other studies with growing beef cattle did report a reduction in DMI with 3-NOP supplementation (Romero-Perez et al., 2014; Vyas et al., 2016), whilst effects of 3-NOP on BW and ADG were absent (Romero-Perez et al., 2014) or limited (Vyas et al., 2016). As such, the response of growing calves of less than 6 mo of age to 3-NOP supplementation in terms of CH_4_ emission, feed intake, and growth has not been completely elucidated yet.

Therefore, the aim of the current study was to evaluate the effect of 3-NOP on CH_4_ emission, feed intake, and ADG for calves from 5 until 8 mo of age. It was hypothesized that 3-NOP supplementation would persistently decrease CH_4_ emission without affecting feed intake and growth performance.

## Materials and Methods

### Study design

The study was conducted from June to October 2022 at the research facility of Wageningen Livestock Research (Dairy Campus, Leeuwarden, the Netherlands), under the Dutch law on Animal Experiments in accordance with European Union Directive 2010/63 and approved by the Animal Welfare Body of Wageningen Research (Lelystad, Netherlands). The study followed a complete randomized block design with 2 dietary treatments and 60 calves that were on average 5 mo of age at the start of the treatment period (5.1 ± 0.11 mo of age; mean ± SD). Each dietary treatment group consisted of 9 females (4 Belgian Blue and 5 Holstein Friesian) and 21 males (all Holstein ­Friesian). The Belgian Blue calves were included in the study due to a limited availability of Holstein Friesian calves of a similar age. Part of the calves (*n* = 17, of which *n* = 7 for the dietary control group and *n* = 10 for the dietary treatment group) were born and housed on Dairy Campus until the start of the study, whereas the other calves (*n* = 43, of which *n* = 23 for the dietary control group and *n* = 20 for the dietary treatment group) were purchased from several local farms and housed at a specialized calf rearing quarantine facility in accordance with the Dairy Campus procedures from 2 wk of age to 4 mo of age. This procedure was followed to ensure a minimum age difference between all calves (i.e. 2 wk), which would not have been possible when enrolling calves from Dairy Campus alone, whilst ensuring the strict health status requirements of Dairy Campus.

The study consisted of an acclimatization period, a baseline measurement period, and a treatment period. In total, 70 calves were enrolled in the acclimatization period, which lasted for 3 wk. During the acclimatization period, the calves could adapt to the new barn and learn to use the GreenFeed system (C-Lock Inc., Rapid City, SD) and the automated Insentec feed bins (Roughage Intake Control (RIC) system, Hokofarm Group BV, Marknesse, Netherlands). At the end of the acclimatization period, 60 calves were selected based on their ability to use the feed bins and GreenFeed system. These 60 calves (192 ± 17.0 kg of BW) were blocked in pairs by sex, breed, and BW, and within block randomly assigned to 1 of the 2 dietary treatments. The acclimatization period was followed by a baseline measurement period of 2 wk. After the baseline measurement period, the treatment period started, consisting of 12 wk during which the calves received 1 of the 2 dietary treatments.

During the entire study, calves were loose housed together in 1 pen consisting of a slatted floor at the feed aisle and the remainder being a deep litter area. The deep litter area was filled with fresh straw once weekly. The calves had access to 2 GreenFeed systems and 12 feed bins and had free access to a water trough.

### Feeding and dietary treatments

During the baseline measurement period, all calves received the same acclimatization diet, fed as a partially mixed ration (**PMR**). This PMR was a mixture of roughage and concentrates (Tables 1 and 2), but not representing the complete diet as the calves received a small amount of concentrates (i.e. bait) in the GreenFeed system as well (see below for more details). The PMR was formulated to reflect a diet commonly used for rearing calves. After the baseline measurement period, the calves were assigned to 1 of the 2 dietary treatments: (1) a PMR supplemented with a placebo (i.e. silicon dioxide and 1,2 propanediol; control [**CTL**]) with a target 3-NOP dose of 0 mg/kg dry matter (**DM**), or (2) a PMR supplemented with 3-NOP (i.e. 10% 3-NOP on silicon dioxide and 1,2-propanediol) with a target 3-NOP dose of 175 mg/kg DM in the PMR, which corresponded with a target dose of 150 mg/kg DM on complete ration level (including the non-supplemented GreenFeed bait). The use of 3-NOP in animal feed was approved by the Veterinary Drugs Directorate Division (Utrecht, Netherlands) prior to the start date.

**Table 1.** Chemical composition (in g/kg DM, unless stated otherwise) of the individual feed ingredients

	Corn silage	Alfalfa	Concentrate			Soybean meal	GreenFeed bait^4^
			Acclimatization diet^1^	CTL^2^	3-NOP^3^		
**DM, g/kg**	355	914	889	884	884	877	883
**OM**	957	899	926	926	921	935	936
**CP**	65	172	149	135	143	464	122
**Crude Fat**	28	24	41	34	33	25	31
**Gross energy, MJ/kg DM**	18.6	16.9	16.3	16.4	16.3	17.3	15.6
**NDF**	354	387	283	280	276	125	337
**ADF**	207	291	159	160	156	63	204
**ADL**	9.8	62	44	43	42	1	14
**Starch**	380	16	278	268	263	21	116
**Sugar**	0	51	33	31	30	100	92

1 Ingredient composition (g/kg DM): palm kernel flakes = 297, corn = 284, wheat semolina = 120, wheat = 101, rumen protected rapeseed meal (Mervobest, NuScience) = 75, lupine = 41, CaCO_3_ = 24, alfalfa = 20, citrocol = 20, NaCl = 10, MgO = 5, and mineral premix = 2.

2 Ingredient composition (g/kg DM): palm kernel flakes = 282, corn = 270, wheat semolina = 113, wheat = 104, rumen protected rapeseed meal (Mervobest, NuScience) = 71, lupine = 47, alfalfa = 19, sugar beet pulp = 19, NaCl = 9, mineral premix = 8, and placebo = 58.

3 Ingredient composition (g/kg DM): palm kernel flakes = 282, corn = 270, wheat semolina = 113, wheat = 104, rumen protected rapeseed meal (Mervobest, NuScience) = 71, lupine = 47, alfalfa = 19, sugar beet pulp = 19, NaCl = 9, mineral premix = 8, and 3-NOP = 58.

4 Ingredient composition (g/kg DM): sugar beet pulp = 300, soybean meal = 221, corn gluten meal = 87, wheat = 72, alfalfa = 70, sunflower kernel meal = 70, molasses = 50, barley = 50, corn = 49, rapeseed meal = 21, NaCl = 10.

**Table 2.** Ingredient and chemical composition (in g/kg DM, unless stated otherwise) of the PMR diets, where GreenFeed bait is not included in the calculation^1^

	Acclimatization diet	CTL	3-NOP
**Diet composition**			
** Corn silage**	658	656	656
** Alfalfa**	168	168	168
** Acclimatization diet concentrate**	28	0	0
** CTL concentrate**	0	31	0
** 3-NOP concentrate**	0	0	31
** Soybean meal**	138	137	137
** Minerals**	8	8	8
**Chemical composition**			
** DM, g/kg**	364	367	367
** OM**	936	936	936
** CP**	140	139	139
** Crude Fat**	27	27	27
** Gross energy, MJ/kg DM**	17.9	17.9	17.9
** NDF**	323	323	323
** ADF**	199	198	198
** ADL**	18	18	18
** Starch**	264	263	263
** Sugar**	23	23	23

1 CTL = control diet with 0 mg 3-NOP/kg DM, 3-NOP = 3-NOP diet with 150 mg 3-NOP/kg DM.

Feeding rate of all diets was adjusted daily to yield refusals equal to a target of 10% of intake. Fresh PMR was prepared once daily, by using a Trioliet feed mixing robot (Trioliet feeding technology, Oldenzaal, Netherlands) and delivered once daily to the feed bins. Prior to offering new feed, feed refusals were removed from the feed bins. Calves had access to all feed bins with the appropriate treatment diet, resulting in 60 calves having access to 12 feed bins during the baseline measurement period or each group of 30 calves having access to their allocated 6 feed bins during the treatment period, always representing 5 calves per feed bin. The calves also had access to 2 GreenFeed systems, where they received a sweet and palatable GreenFeed bait (Table 1) for enticement and to encourage the calves to maintain a suitable head position for accurate measurements. This GreenFeed bait was provided with a maximum of 8 so-called cup drops per visit, 1 cup drop per 30 s, and 44.0 ± 1.05 g of bait per cup drop, with a visit interval of 4 h and a maximum of 4 visits/d.

### Measurements

Individual DMI of the PMR was automatically recorded at each visit of the animal to the feed bin. The amount of GreenFeed bait received by each animal was also recorded in terms of the amount of cup drops of feed that the animal received at each visit. On a weekly basis throughout the study, the amount of bait delivered in each cup drop was weighed and tested for both GreenFeed systems, to ensure accuracy on the calculation of DMI. Total DMI was calculated by adding the DMI of the PMR to the DMI of the GreenFeed bait on a daily basis.

The individual BW of each animal was manually recorded weekly by placing them on a weighing scale within the barn (Welvaarts Weegsystemen BV, ‘s-Hertogenbosch, Netherlands). The following variables from these recordings were used in data analyses: weekly BW, initial BW (single measure at 1 week after start of the acclimatization period), final BW (single measure at the end of the treatment period), BW gain (final BW—initial BW), and ADG (BW gain/days[final—initial]). The gain-to-feed ratio was calculated by dividing ADG (kg) by average daily total DMI (kg).

Gas emissions (CH_4_, hydrogen [**H_2_**], and carbon dioxide [**CO_2_**]) were measured on individual animal basis by using the GreenFeed system as described by van Gastelen et al. (2022). All calves had free access to 2 GreenFeed systems. Of all GreenFeed visits during the treatment period (12 weeks; 15,788 visits), 42% were in GreenFeed system A and 58% in GreenFeed system B. Both GreenFeed systems were equally visited across treatments, where 51% and 49% of the visits to GreenFeed system A were for 3-NOP and CTL calves, respectively. For GreenFeed system B, 53% and 47% were for 3-NOP and CTL calves, respectively. The average CO_2_ recovery was 99.4% (± 3.1%). The yield of CH_4_, H_2_, and CO_2_ was calculated using the daily DMI (PMR + GreenFeed bait) averaged for the respective wk and was expressed as g/kg total DMI. Similarly, methane intensity was calculated on a BW basis as g CH_4_/kg BW.

### Feed samples and chemical analysis

During the study, corn silage samples were collected daily and dry ration component samples (i.e. alfalfa, concentrates, and soybean meal) were collected weekly to determine DM concentration as described by Abrahamse et al. (2008). This information was subsequently used to determine how much of each ration component as fresh product was required to prepare the diets. Hence, on a weekly basis, the formulations of the diets were adjusted according to the DM results of the samples collected in the previous week. Furthermore, samples of the separate PMR components (i.e. corn silage, alfalfa, concentrates, and soybean meal) and GreenFeed bait, were collected weekly and stored at −20°C pending analysis. These feed samples were sent to the laboratory of Animal Nutrition (Wageningen University & Research, Wageningen, the Netherlands) for analyses and used, in combination with the Trioliet feed mixing robot data, to determine dietary composition and DMI. Additionally, a representative sample of PMR was also collected from each treatment group at four different timepoints in week 3, 6, 9 and 12 of the treatment periods, and stored at −20°C pending analysis. The PMR samples were sent to Global R&D Analytics NIC-RD/A of dsm-firmenich (Kaiseraugst, Switzerland) for the analysis of 3-NOP concentration, according to the procedure described by van Gastelen et al. (2020).

All collected feed samples were thawed at room temperature. Corn silage was subsequently freeze-dried until constant weight and ground to pass a 1-mm screen by using a cross-beater mill (Peppink 100AN, Olst, the Netherlands). All other samples were ground to pass a 1-mm screen by using an ultra-centrifugal mill (Retsch ZM200, Retsch GmbH, Haan, Germany). The samples were subsequently analyzed for DM, ash, nitrogen (**N**), starch, reducing sugars (i.e. all carbohydrates with reducing properties and soluble in 40% ethanol; except for corn silage), crude fat, neutral detergent fiber (**NDF**), acid detergent fiber (**ADF**), and acid detergent lignin (**ADL**) as described by Klop et al. (2016). The DM concentration was determined by drying at 103 °C until constant weight (ISO 6496; International Organization for Standardization, 1999a). Ash was determined after combustion at 550 °C (ISO 5984; International Organization for Standardization, 2002). Hydrolysis with HCl and extraction with light petroleum was used to determine crude fat (ISO 6492; International Organization for Standardization, 1999b). Starch was determined enzymatically (ISO 15914; International Organization for Standardization, 2004). The NDF was analyzed according to a modified method of van Soest et al. (1991) after pretreatment with α-amylase and protease, but without sodium sulfite. Concentrations of ADF and ADL were determined according to van Soest et al. (1991) and Robertson and van Soest (1981), respectively. Sugar analysis was carried out as described by van Vuuren et al. (1993) using a 40% ethanol solution, with modifications to this method as described by Abrahamse et al. (2008). Bomb calorimetry (ISO 9831; International Organization for Standardization, 1998) was used to determine gross energy. Crude protein was calculated as N × 6.25, where N was determined using the Kjeldahl method with cupric sulfate (CuSO_4_) as catalyst (ISO 5983; International Organization for Standardization, 2005). The N concentrations in the corn silage was determined in fresh material, which were deproteinized by the addition of 10% (wt/vol) trichloroacetic acid solution followed by centrifugation. Subsequently, indophenol blue was formed using the Berthelot reaction with phenol and hypochlorite in an alkaline solution, which was determined spectroscopically at 623 nm.

### Statistical analysis

One male calf needed to be removed from the study after week 6 of the treatment period due to aggressive behavior and data from one other male calf were excluded after week 6 of the treatment period due to health issues (coccidiosis) compromising his DMI and BW gain. Both animals were from the 3-NOP treatment group. Data for the 2 steers that were removed from the study were included up until their removal.

Statistical analysis was performed using SAS version 9.4 (SAS Institute Inc., Cary, NC). Data were averaged for the baseline measurement period and for treatment wk 1–3, 4–6, 7–9 and 10–12. The baseline measurement period was used to calculate a covariate mean for all variables, which was added in the statistical models as baseline measurement. A repeated measurements model (PROC MIXED) with animal as the repeated subject was used to test the effects of treatment (CTL or 3-NOP), period (wk 1–3, 4–6, 7–9, and 10–12), their interaction, and the covariate mean on dependent variables related to emissions, visits to the GreenFeed system, feed intake, and BW. When significant treatment × period interactions were detected, treatment effects (CTL vs. 3-NOP) were evaluated within each period to interpret the interaction. Moreover, a linear mixed model (PROC MIXED) was used to test the effects of treatment and, when applicable, the covariate mean on the dependent variables initial BW, final BW, BW gain, ADG, and gain-to-feed ratio. Initial BW was used as the covariate mean for final BW, BW gain, and ADG. All analyses included a random effect of block (defined by sex, breed, and BW; *n* = 30) and animal.

Model residuals were assessed for normality, outliers, and independence. For the number of GreenFeed visits, residuals followed a non-normal distribution. Consequently, this variable was inverse transformed for the statistical analysis, and *P*-values were obtained via bootstrapping. For the repeated measurement models, the covariance structure with the best fit was selected based on the model with the lowest corrected Akaike Information Criterion with a correction for small sample sizes (AICC). Covariance structures considered were Compound Symmetry (CS), Heterogeneous CS (CSH), Unstructured (UN), First Order Autoregressive (AR(1)), Heterogeneous AR(1) (ARH(1)). Values are presented as covariate-adjusted least squares means (**LSM**). Significance of effects was declared at *P* ≤ 0.05 and trends at 0.05 < *P* ≤ 0.10.

## Results

### Dosage of 3-NOP

The 3-NOP dose in the PMR samples of the CTL diet was 0 mg/kg. The 3-NOP dose in the PMR samples of the 3-NOP diet was 145 ± 5.0 mg 3-NOP/kg DM, which was below target (i.e. 175 mg 3-NOP/kg DM). It should also be noted that because the GreenFeed bait was not supplemented with 3-NOP this would have effectively further diluted the dose rate of 3-NOP received on a total DMI basis. On average, over the course of the treatment period, animals in the 3-NOP treatment group consumed 1.05 kg GreenFeed bait/day (DM basis) which would have accounted for 17.5% of their total DMI (6.1 kg/d). Considering the intake of GreenFeed bait, the average level of 3-NOP in the total daily DMI was 121 mg 3-NOP/kg DM on complete ration level, as opposed to the target dose of 150 mg 3-NOP/kg DM.

### Effect of 3-NOP on gas emission

**Figure 1.**
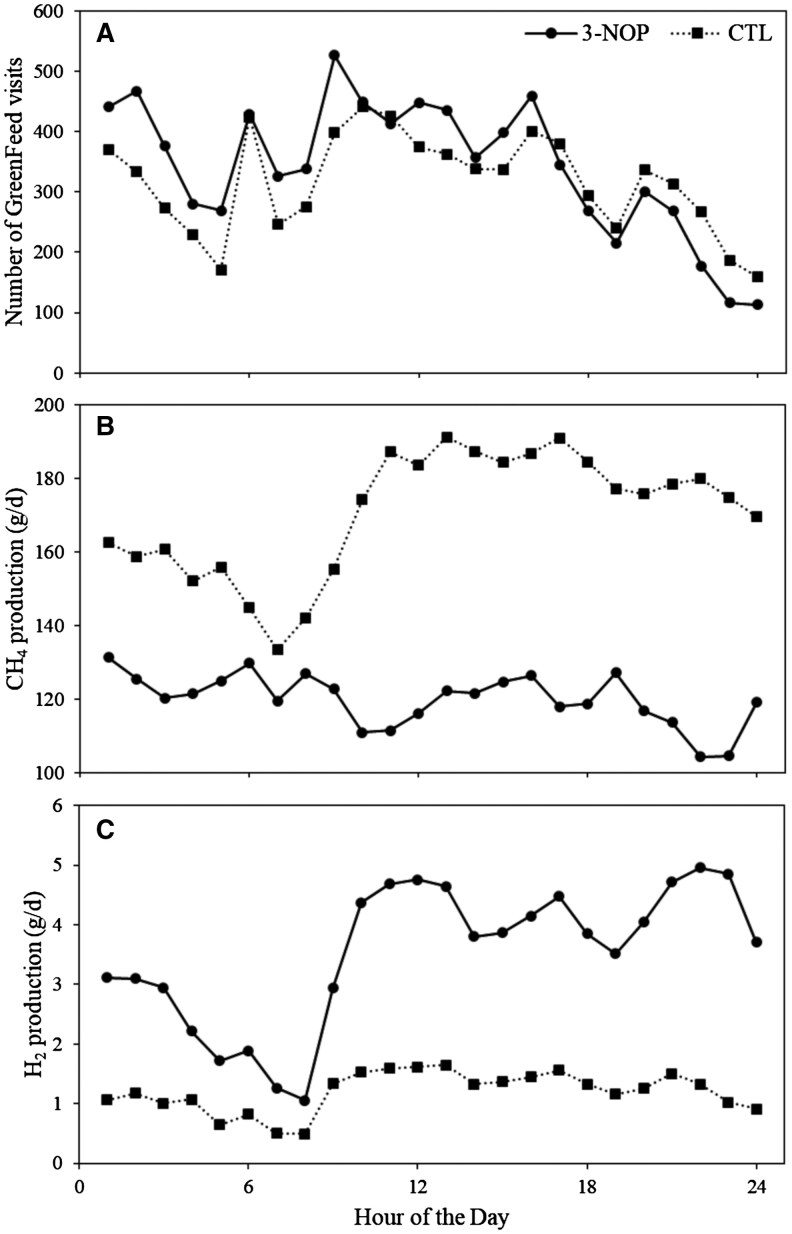
Total number of GreenFeed visits over the complete treatment period per hour (A), average CH_4_ production (g/d) per hour (B), and average H_2_ production per hour (C) of calves from 5 mo of age receiving a diet supplemented with a placebo (CTL) or with 3-nitrooxypropanol (3-NOP). The dashed line with squares represents CTL and the solid line with circles represents 3-NOP.

For the visits to the GreenFeed system, a treatment × period interaction (*P *= 0.05) was observed (Table 3). In every period, calves receiving 3-NOP visited the GreenFeed system more often compared to calves receiving CTL (*P *< 0.01 for all ­periods). Gas emissions measures by the GreenFeed system occurred throughout the full 24-h period (Figure 1A).

**Table 3.** Gas emissions of growing calves from 5 mo of age receiving a diet supplemented with a placebo (CTL) or with 3-nitrooxypropanol (3-NOP) treatment (trt) per period (P; wk 1–3, wk 4–6, wk 7–9, wk 10–12)

	Treatment								SEM	*P*-value		
	Wk 1–3		Wk 4–6		Wk 7–9		Wk 10–12					
	CTL	3-NOP	CTL	3-NOP	CTL	3-NOP	CTL	3-NOP		Trt	P	Trt*P
**n experimental units^1^**	30	30	30	30	30	28	30	28				
**GreenFeed visits, #/d^2^**	3.4^b^	3.6^a^	2.9^b^	3.3^a^	2.8^b^	3.3^a^	2.7^b^	3.2^a^	0.24	0.13	<0.01	0.05
**CH_4_ emission**												
** Production, g/d**	154^a^	106^b^	172^a^	125^b^	183^a^	128^b^	185^a^	128^b^	7.2	<0.01	<0.01	0.04
** Yield, g/kg DMI**	25.1	20.0	26.1	21.8	26.6	21.5	25.9	20.3	1.38	<0.01	<0.01	0.23
** Intensity, g/kg BW**	0.70^a^	0.49^b^	0.70^a^	0.52^b^	0.68^a^	0.49^b^	0.63^a^	0.45^b^	0.029	<0.01	<0.01	0.04
**H_2_ emission**												
** Production, g/d**	1.21^b^	3.69^a^	1.24^b^	3.61^a^	1.33^b^	3.75^a^	1.22^b^	3.25^a^	0.331	<0.01	<0.01	0.02
** Yield, g/kg DMI**	0.20^b^	0.68^a^	0.19^b^	0.62^a^	0.19^b^	0.63^a^	0.17^b^	0.50^a^	0.054	<0.01	<0.01	<0.01
**CO_2_ emission**												
** Production, g/d**	5477	5384	6140^a^	5846^b^	6501^a^	6225^b^	6746^a^	6325^b^	173.1	<0.01	<0.01	<0.01
** Yield, g/kg DMI**	892	1007	936	1020	950	1038	966	993	48.9	<0.01	<0.01	0.11

1 Two animals were removed from the study; data were included up to wk 6.

2 Approved GreenFeed visits only, which were used for gas emission data. Inverse transformed for the statistical analysis, LSM are back-transformed and should be interpreted as medians, *P*-values obtained via bootstrapping.

a,b Values within a row with different superscripts differ significantly within that period (i.e. only when there is a Trt × P interaction) at *P *< 0.01.

**Figure 2.**
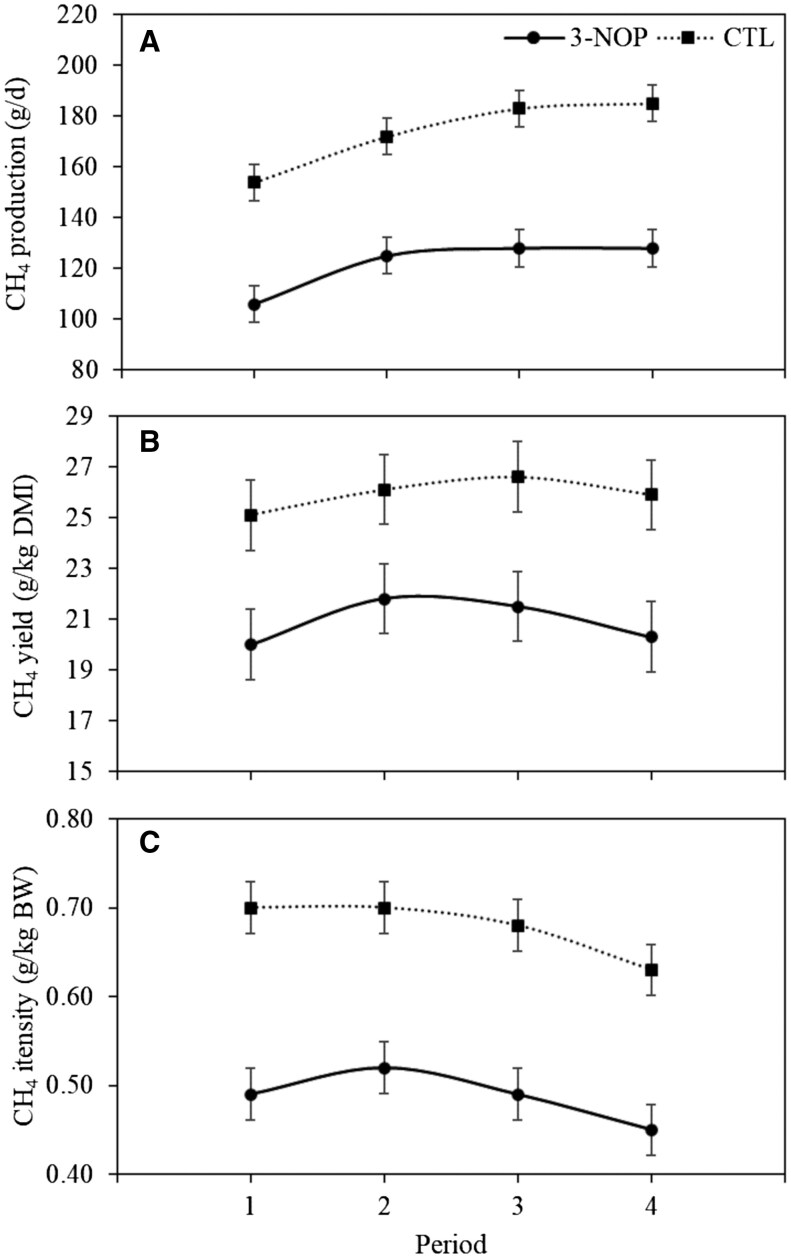
Methane production (panel A), yield (panel B), and intensity (panel C) of calves from 5 mo of age receiving a diet supplemented with a placebo (CTL) or with 3-nitrooxypropanol (3-NOP). Period 1 refers to wk 1–3, period 2 refers to wk 4–6, period 3 refers to wk 7–9, and period 4 refers to wk 10–12. The dashed line with squares represents CTL and the solid line with circles represents 3-NOP. Bars represent standard error.

For the production and intensity of CH_4_, a treatment × period interaction (*P *= 0.04) was observed. In every period, calves receiving 3-NOP had a lower CH_4_ production and intensity compared to calves receiving CTL (*P *< 0.01 for all periods) (­Figure 2). Compared to CTL, CH_4_ production was reduced by 31.3% in wk 1–3, 27.1% in wk 4–6, 30.0% in wk 7–9, and 30.7% in wk 10–12 for 3-NOP. Compared to CTL, CH_4_ intensity was reduced by 30.0% in week 1–3, 25.8% in wk 4–6, 27.9% in wk 7–9, and 28.6% in wk 10–12 for 3-NOP. Over the entire treatment period, the 3-NOP calves had a reduced CH_4_ production and intensity (CTL vs. 3-NOP; 174 ± 2.0 g/d vs. 122 ± 2.0 g/d, *P *< 0.01 and 0.68 ± 0.008 g/kg BW vs. 0.49 ± 0.008 g/kg BW, *P *< 0.01) compared to CTL. As such, the 3-NOP calves had a reduction of 29.8% for CH_4_ production and 27.9% for CH_4_ intensity. No interaction between treatment and period was observed for CH_4_ yield. Over the entire treatment period, calves receiving 3-NOP had a reduced CH_4_ yield (CTL vs. 3-NOP; 25.9 ± 0.38 g/kg total DMI vs. 20.9 ± 0.38 g/kg total DMI, *P *< 0.01) compared to CTL, corresponding to a 19.4% reduction. When investigating the CH_4_ production over a 24-h period, fluctuations existed where especially during the day CH_4_ production was higher, mainly for the CTL group (Figure 1B).

For H_2_ production and yield, a treatment × period interaction (*P *= 0.02 and *P *< 0.01, respectively) was observed. In every period, calves receiving 3-NOP had a greater H_2_ production and yield compared to calves receiving CTL (*P *< 0.01 for all periods). Compared to CTL, H_2_ production was increased by 205% in wk 1–3, by 191% in wk 4–6, by 182% in wk 7–9, and by 166% in wk 10–12 for 3-NOP. Compared to CTL, H_2_ yield was increased by 240% in wk 1–3, by 226% in wk 4–6, by 232% in wk 7–9, and by 194% in wk 10–12 for 3-NOP. Over the entire treatment period, the 3-NOP calves had an increased H_2_ production and yield (CTL vs. 3-NOP; 1.25 ± 0.095 g/d vs. 3.57 ± 0.095 g/d, *P *< 0.01 and 0.19 ± 0.015 g/kg DMI vs. 0.61 ± 0.015 g/kg DMI, *P *< 0.01) compared to CTL. As such, the 3-NOP calves had an increase of 185% for H_2_ production and 221% for H_2_ yield. When investigating the H_2_ production over a 24-h period, fluctuations existed, where especially during the day H_2_ production was higher, mainly for the 3-NOP group (Figure 1C).

For the production of CO_2_, a treatment × period interaction (*P *< 0.01) was observed. The production of CO_2_ did not differ between CTL and 3-NOP in the first period (week 1–3) but was higher for CTL compared to 3-NOP in the three following periods (i.e. week 4–6, 7–9, and 10–12, *P *< 0.01). Over the entire treatment period, calves receiving 3-NOP had a reduced CO_2_ production of 4.4% (CTL vs. 3-NOP; 6216 ± 45.3 g/d vs. 5945 ± 45.7 g/d, *P *< 0.01), but an increased CO_2_ yield of 8.4% (CTL vs. 3-NOP; 936 ± 10.6 g/kg total DMI vs. 1014 ± 10.8 g/kg total DMI, *P *< 0.01).

### Effect of 3-NOP on feed intake and body weight

For DMI of GreenFeed bait, a treatment × period interaction (*P *< 0.01) was observed, where during all periods, calves receiving 3-NOP had a greater DMI of GreenFeed bait compared to calves receiving CTL (Table 4) (*P *< 0.01 for all comparisons between the groups within the same period). Over the entire treatment period, calves receiving 3-NOP had a lower DMI of PMR compared to calves receiving CTL (5.0 ± 0.07 kg/d vs. 5.9 ± 0.07 kg/d, respectively, *P *< 0.01). Moreover, the calves in the 3-NOP group had a lower total DMI over the entire treatment period compared to the calves in the CTL group (6.1 ± 0.08 kg/d vs. 6.9 ± 0.07 kg/d, *P *< 0.01).

**Table 4.** Dry matter intake and BW of growing calves from 5 mo of age receiving a diet supplemented with a placebo (CTL) or with 3-nitrooxypropanol (3-NOP) treatment (trt) per period (P; wk 1–3, wk 4–6, wk 7–9, wk 10–12)

	Treatment								SEM	*P*-value		
	Wk 1–3		Wk 4–6		Wk 7–9		Wk 10–12					
	CTL	3-NOP	CTL	3-NOP	CTL	3-NOP	CTL	3-NOP		Trt	P	Trt*P
**n experimental units^1^**	30	30	30	30	30	28	30	28				
**Total DMI, kg/d**	6.3	5.5	6.7	5.9	7.0	6.2	7.5	6.7	0.30	<0.01	<0.01	0.99
**GreenFeed bait DMI, kg/d^2^**	1.09^b^	1.14^a^	0.93^b^	1.04^a^	0.91^b^	1.04^a^	0.86^b^	0.99^a^	0.051	<0.01	<0.01	<0.01
**Partially mixed ration DMI, kg/d**	5.20	4.36	5.77	4.81	6.08	5.12	6.64	5.73	0.297	<0.01	<0.01	0.83
**BW, kg^3^**	220^a^	216^b^	245^a^	240^b^	271^a^	263^b^	295^a^	284^b^	4.1	<0.01	<0.01	0.05

1 Two animals were removed from the study; data were included up to wk 6.

2 Based on intake of GreenFeed bait in all GreenFeed visits, also when the visit was not approved (i.e., not suitable for gas emission data).

3 For the analysis of BW of the 3-NOP group, in wk 4–6 *n* = 29 because the animal was removed before BW was measured in wk 6, although sufficient gas data were collected.

a,b Values within a row with different superscripts differ significantly within that period (i.e. only when there is a Trt × P interaction) at *P *< 0.01.

For BW, a treatment × period interaction (*P *= 0.05) was observed. Over the entire treatment period, calves in the 3-NOP group had a lower BW compared to CTL (251 ± 1.2 kg vs. 258 ± 1.2 kg, *P *< 0.01), and this difference in BW between 3-NOP and CTL was present in all 4 periods. The 3-NOP group and the CTL group did not differ in initial BW, but calves in the 3-NOP group had a lower final BW compared to CTL (*P *< 0.01, Table 5). As a result, calves in the 3-NOP group also had a lower BW gain and lower ADG during the complete treatment period compared to CTL (*P *< 0.01). Despite the observed difference in DMI and ADG between the 3-NOP and CTL group, gain-to-feed ratio was not affected by 3-NOP supplementation.

**Table 5.** Body weight, BW gain, and gain-to-feed ratio of growing calves from 5 mo of age over the full 12-week period in which a diet supplemented with a placebo (CTL) or with 3-nitrooxypropanol (3-NOP) was fed

	Treatment		SEM	*P*-value
	CTL	3-NOP		Trt
**n experimental units^1^**	30	28		
**Initial BW, kg^2^**	203.1	202.4	4.96	0.83
**Final BW, kg**	300.5^a^	290.3^b^	3.22	<0.01
**BW gain, kg**	98.2^a^	87.9^b^	3.22	<0.01
**Average daily gain, kg**	1.17^a^	1.05^b^	0.04	<0.01
**Gain-to-feed ratio, kg BW gain/kg DMI**	0.173	0.175	0.0061	0.61

1 Two animals in the 3-NOP group were removed from the study.

2 For the analysis of the initial BW of the 3-NOP group, *n* = 29: excluded the datapoint of only 1 animal, because data were excluded from this animal in the latter part of the study due to health issues after wk 6. For the other variables in this table, both animals that were removed after wk 6 are excluded (i.e., *n* = 28).

a,b Values within a row with different superscripts differ significantly at *P *< 0.05.

## Discussion

The objective of this study was to determine the effect of 3-NOP on CH_4_ emission, feed intake, and ADG for calves from 5 until 8 mo of age. Hence, we will focus specifically on the 3-NOP effect and the interaction between 3-NOP and period when discussing the results. When treatment × period interactions were observed, specific focus will be on the difference or lack of difference between CTL and 3-NOP within each period when discussing the results.

### Methane emission

It was hypothesized that 3-NOP supplementation would decrease CH_4_ emission and that this effect would be persistent over time. Indeed, in the current study, supplementation with 3-NOP reduced CH_4_ production, yield, and intensity by 29.8%, 19.4%, and 27.9% compared to CTL, respectively, with relatively minor differences between periods (< 5% units). These reduction percentages were lower than what could be expected based on the meta-analysis by Orzuna-Orzuna et al. (2024; production: 34.9% and yield: 29.6%), but slightly higher than what could be expected based on the meta-analysis by Dijkstra et al. (2018; production: 22.2% and yield: 17.1%, after adjustment for the effects of 3-NOP dose and dietary NDF concentration.

Despite the persistent CH_4_ reduction by 3-NOP, an interaction effect between treatment and period was observed for both CH_4_ production and the intensity. However, within each period, both CH_4_ production and intensity were lower for calves receiving 3-NOP compared to CTL without declining reduction percentages, suggesting a persistent CH_4_ reducing effect of 3-NOP (Figure 2). The animals enrolled were growing calves from 5 mo of age, which implies that the DMI and BW of the calves increased over the course of the study, and as expected, CH_4_ production also increased. For the CTL calves, CH_4_ production increased relatively more during the treatment period (+ 31 g/d) than that of the 3-NOP calves (+ 22 g/d), which may to a certain extent explain the observed interaction between treatment and period for CH_4_ production. Due to a similar increase in CH_4_ production and DMI over time, CH_4_ yield was stable over time for both CTL and 3-NOP calves, explaining the lack of interaction for CH_4_ yield. In contrast, as the increase in BW over time was slightly greater compared to the increase in CH_4_ production, the CH_4_ intensity declined only slightly over time. The greater increase in BW for calves receiving CTL compared to 3-NOP may have resulted in the interaction between treatment and period. The meta-analysis by Orzuna-Orzuna et al. (2024) demonstrated that the daily CH_4_ emission decreased for calves receiving 3-NOP, regardless of the supplementation period. In line with these results, in the current study, the reductions of CH_4_ production, yield, and intensity did not decrease over the course of the treatment duration, indicating a persistent efficacy of 3-NOP on the reduction of CH_4_ emission, and no adaptation to 3-NOP supplementation over the (relatively short) treatment period of 12 wk.

### Hydrogen and carbon dioxide emission

The H_2_ production was 1.9-fold greater and H_2_ yield was over 2.2-fold greater in the calves receiving 3-NOP compared to CTL. This increase in H_2_ is often observed when methanogenesis—the main H_2_ sink—is inhibited via 3-NOP supplementation (e.g., Vyas et al., 2016; Alemu et al., 2021). However, the observed interactions for both H_2_ production and yield show that H_2_ emission was always greater for 3-NOP compared to CTL, but that the percentage increase in both H_2_ production and yield upon 3-NOP supplementation decreased over the course of the treatment period. This reduction in H_2_ emission was unrelated to the percentage reduction for both CH_4_ production and yield upon 3-NOP supplementation. This suggests that with time of 3-NOP application, H_2_ may have been redirected into an alternative H_2_ sink, because H_2_-consuming pathways are often upregulated when ruminal H_2_ concentration is increasing (Jansen, 2010), and to propionate and butyrate at the expense of acetate delivering less H_2_. Although not measured in the current study, this increase in propionate and butyrate upon 3-NOP supplementation has been observed in other studies with corn silage-based diets (e.g., Romero-Perez et al., 2014; Haisan et al., 2017).

The measured increase in H_2_ emission upon 3-NOP supplementation was lower than the expected stoichiometric amount that is involved in the decrease of CH_4_ production. This lower-than-expected recovery of H_2_ that was spared from utilization with methanogenesis was also observed by Hristov et al. (2015) and van Gastelen et al. (2020, 2022), and suggests that the secondary effects of 3-NOP include a redirection of H_2_ to alternative H_2_ sinks, with a redirection of the ruminal fermentation profile toward propionate fermentation as a H_2_ sink being a likely consequence (Romero-Perez et al., 2014; Haisan et al., 2017). Also the measurement techniques that was used in the current study may explain partially the lower-than-expected recovery of H_2_. van Lingen et al. (2017) for example showed that H_2_ emissions were a factor 100 greater in the first hour after a meal. The GreenFeed system is a spot sampling device that records gas emissions at various timepoints throughout the day, and as a result, may have missed this major postprandial peak in H_2_ emission. de Mol et al. (2024) demonstrated that the level of H_2_ measured by the GreenFeed system decreased when the time interval between a preceding meal and the moment of H_2_ measurement increased. If the calves of the current study did not visit the GreenFeed system shortly after consuming a meal at the feed bin, the large peak in H_2_ emission following a meal would not be accounted for in the daily estimate and H_2_ emission could have been underestimated. van Lingen et al. (2023) demonstrated that a sampling interval of 2 h was required of dairy cows fed twice daily ad libitum to obtain accurate daily H_2_ emission values that did not differ from continuous measurements in climate respiration chambers. Although the GreenFeed measurements in the current study covered the full 24-h period (Figure 1A), the GreenFeed visiting interval of 4 h was considerably larger than the interval advised by van Lingen et al. (2023).

The observed interaction for CO_2_ production shows that calves receiving 3-NOP had a lower CO_2_ production compared to the CTL calves from wk 4 onwards. This is likely related to the slower growth rate of the 3-NOP supplemented calves compared to the CTL calves, as a consequence of the decreased DMI (see below for more details). Calves receiving 3-NOP had an increased CO_2_ yield compared to CTL, which has been observed before in dairy cows (e.g., van Gastelen et al., 2024). This increased CO_2_ yield is likely partially caused by the lower DMI in the 3-NOP supplemented animals, to the slower growth rate, and to a small extent by a lower CO_2_ utilization due to less CH_4_ production.

### Feed intake and body measures

Supplementation with 3-NOP resulted in a reduction of DMI (11.6%). This reduction in total DMI is opposite to the GreenFeed bait DMI, which was—despite the observed interaction—higher for the calves receiving 3-NOP compared to the CTL calves in all periods. This is supported by the increased number of GreenFeed visits of calves receiving 3-NOP, although the reason for this discrepancy between total DMI and bait DMI remains unclear. Thus, the reduction in DMI of calves receiving 3-NOP was driven by a reduction in PMR intake. Moreover, the reduction in DMI started as soon as the treatment started for the calves receiving 3-NOP. Some other studies on growing calves also reported a reduction in DMI upon supplementation with 3-NOP (Romero-Perez et al., 2014; Vyas et al., 2016). In these 2 studies, as well as in the current study, starch-rich diets (i.e. >250 g/kg DM) were fed. Kirwan et al. (2024) did not observe an effect on DMI upon 3-NOP supplementation in growing beef cattle fed a low starch diet (i.e. 189 g/kg DM). The diet composition and the general effect of 3-NOP supplementation on ruminal volatile fatty acids (**VFA**) and H_2_ emission may have played a role in the observed decrease in DMI upon 3-NOP supplementation in the present study. When fermentable carbohydrates, such as starch, are metabolized in the rumen, VFA are produced, including propionate. Hence, a high starch diet results in increased ruminal concentration of propionate compared to a low starch diet (e.g., Ellis et al., 2008). According to the meta-analysis by Orzuna-Orzuna et al. (2024), ruminal concentration of propionate increases upon 3-NOP supplementation. Therefore, although not measured in the present study, it is likely that the calves (both CTL and 3-NOP) already had a relatively high ruminal concentration of propionate due to dietary starch concentration, and that this concentration increased even more for the 3-NOP treated calves. Increased production of VFA accompanied with increased molar proportions of propionate could result in an increased portal vein propionate concentration, which can promote satiety, resulting in a decreased DMI (Allen, 2000; Stocks and Allen, 2012). However, the decrease in DMI could also indicate that the rumen microbiota of the calves receiving 3-NOP may not have been able to cope with the excess of the H_2_ that was likely formed because of inhibiting methanogenesis, as an increased ruminal partial pressure of H_2_ can be expected to inhibit the metabolism of rumen microorganisms (McAllister and Newbold, 2008; Morgavi et al., 2010). Although such an inhibitory effect of H_2_ cannot be excluded, it is worth remembering that normal postprandial fluctuations in rumen H_2_ are much larger than the increase in H_2_ emission observed upon 3-NOP supplementation (van ­Lingen et al., 2017).

The lower BW gain (i.e. difference initial and final BW) for 3-NOP calves compared to the CTL calves is likely caused by the lower DMI. A lower DMI means that there is less energy available for growth. Moreover, a lower DMI results in less feed in the gastrointestinal tract, per definition resulting in a lower BW. Supplementation with 3-NOP did not affect gain-to-feed ratio, indicating that any reduction in BW was a direct result of a reduction in DMI. The interaction observed for BW indicates that the difference in BW between CTL and 3-NOP calves is increasing over time (from no difference in BW at start of trial to 4 kg difference in period 1 to 10 kg difference at the start of the trial). In previous studies, BW of calves receiving 3-NOP was unaffected (Kirwan et al., 2024), even when DMI was reduced upon 3-NOP supplementation (Romero-Perez et al., 2014; Vyas et al., 2016; Orzuna-Orzuna et al., 2024). This suggests that younger calves with a lower BW, as were enrolled in the present study, may be more sensitive to reductions in DMI, as they have higher relative energy requirements for growth and less body reserve capacity to buffer intake fluctuations.

## Conclusion

Feeding 121 mg 3-NOP/kg DM on complete ration level to growing calves over a 12 wk period persistently reduced enteric CH_4_ production (g/d) by 29.8%, yield (g/k DMI) by 19.4%, and intensity (g/kg BW) by 27.8%. This was accompanied with a 2-fold increase in H_2_ emission, a lower BW gain (−10.3 kg over 12-wk trial period), and a lower total DMI (−0.8 kg/d). Despite the decreased DMI and growth performance, gain-to-feed ratio (kg BW gain/kg DMI) did not differ between the treatment groups. It can be concluded that 3-NOP persistently decreases CH_4_ emission in growing beef calves from 5 to 8 mo of age, but that it takes a longer period of time to grow the cattle to a similar body weight due to the decreased growth performance.

## Abbreviations:

ADG: average daily gain
ADF: acid detergent fiber
ADL: acid detergent lignin
BW: body weight
CH_4_: methane
CO_2_: carbon dioxide
CTL: control diet with placebo concentrate
DM: dry matter
DMI: dry matter intake
GHG: greenhouse gas
H_2_: hydrogen
N: nitrogen
NDF: neutral detergent fiber
PMR: partial mixed ration
LSM: least squares means
VFA: volatile fatty acid
3-NOP: 3-nitrooxypropanol
